# Self-identified Obese People Request Less Money: A Field Experiment

**DOI:** 10.3389/fpsyg.2016.01454

**Published:** 2016-09-23

**Authors:** Antonios Proestakis, Pablo Brañas-Garza

**Affiliations:** ^1^Health, Consumers and Reference Materials, Joint Research Centre, European CommissionIspra, Italy; ^2^Economics Department, Business School, Middlesex University LondonLondon, UK

**Keywords:** discrimination, obesity, weight-bias, in-group devaluation, system justification theory, wage-gap

## Abstract

Empirical evidence suggests that obese people are discriminated in different social environments, such as the work place. Yet, the degree to which obese people are internalizing and adjusting their own behavior as a result of this discriminatory behavior has not been thoroughly studied. We develop a proxy for measuring experimentally the “self-weight bias” by giving to both self-identified obese (*n* = 90) and non-obese (*n* = 180) individuals the opportunity to request a positive amount of money after having performed an identical task. Consistent with the System Justification Theory, we find that self-identified obese individuals, due to a preexisting *false consciousness*, request significantly lower amounts of money than non-obese ones. A within subject comparison between self-reports and external monitors' evaluations reveals that the excessive weight felt by the “self” but not reported by evaluators captures the self-weight bias not only for obese but also for non-obese individuals. Linking our experimental results to the supply side of the labor market, we argue that self-weight bias, as expressed by lower salary requests, enhances discriminatory behavior against individuals who feel, but may not actually be, obese and consequently exacerbates the wage gap across weight.

## 1. Introduction

Obesity is a salient appearance characteristic, which can severely stigmatize individuals and provoke various forms of prejudice and discrimination in several areas, including the workplace, school, interactions with health professionals and other social settings (see Puhl and Heuer, 2009 for an extensive review). Numerous empirical studies have reported the negative effects of obesity on wages and employment rates (Cawley, 2004, 2007; Cawley and Danziger, 2005; Garcia and Quintana-Domeque, 2006; Brunello and D'Hombres, 2007; Han et al., 2009). For instance, Cawley (2004) estimated that for white females, an increase of 64 pounds above average weight was associated with a 9% decrease in wages. Han et al. (2009) found that the negative relationship between the BMI and wages is larger in occupations requiring social interactions and across older people. Brunello and D'Hombres (2007) observed that a 10% increase in the average BMI reduces the hourly wages of males by 1.9% and of females by 3.3% while Garcia and Quintana-Domeque (2006) reported a negative correlation between wages and obesity, ranging from −2 to −10% but only for women. Although weaker, the negative effects of obesity hold even when more complex measures (which are based on bioelectrical impedance analysis, e.g., total or percent body fat, fat-free mass, etc.) of obesity are employed (Burkhauser and Cawley, 2008; Johansson et al., 2009; Wada and Tekin, 2010). Evidence on discrimination attributed to obesity can also be found in experimental psychology studies (see Roehling et al., 2008 meta-analysis on weight-discrimination). All in all, those papers showed that overweight job applicants and employees were evaluated more negatively and had worse employment outcomes compared to their non-overweight counterparts.

Unlike the bias against other minority groups (e.g., racial, ethnic, religious, etc.), negative attitudes toward overweight individuals are somehow socially accepted and even encouraged, making the stigma of obesity one of the most pervasive and persistent (Wang et al., 2004). Social Identity Theory (Tajfel and Turner, 1979) gives a plausible explanation about inter-group discrimination; distinct groups are more likely to view in-group members in a more positive light and out-group people more negatively [a result which is also experimentally confirmed, e.g., (Bernhard et al., 2006; Chen and Li, 2009); etc. and also introduced into the economic analysis in the seminal study by Akerlof and Kranton (2000)]. However, this theory does not explain in-group, anti-fat attitudes (i.e., negative attitudes and stereotypes about obese people at both the explicit and implicit level) which were documented in several empirical studies (e.g., Rudman et al., 2002; Wang et al., 2004; Crandall and Reser, 2005; Schwartz et al., 2006) and described by the System Justification (Jost and Banaji, 1994) and Social Dominance (Sidanius and Pratto, 2004) theories. More recent studies also make the crucial distinction between intra-group anti-fat attitudes (overweight individuals toward other overweighted individuals) and the internalization of weight bias toward the “self” (Puhl et al., 2007; Durso and Latner, 2008). Along these lines, we use the term “self-weight bias” to describe the internalized weight bias of overweight people toward themselves.

We hypothesize that because of the self-weight bias, obese participants will respond differently to a stimulus related to a compensation for a given task by *claiming less money for themselves*. The concept of “false consciousness” (Elster, 1982; Cunningham, 1987; Eagleton, 1991)—also central in the System Justification Theory (Jost and Banaji, 1994; Jost, 2011)—provides good theoretical grounds for our hypothesis. Obese individuals, like other marginalized groups, may develop a differential attitude due to false consciousness: *the tendency on the part of marginalized group members to implicitly accept society's negative orientations toward their group as justification for their subordinate status* (Rudman et al., 2002). As noted before, many studies have already documented society's negative orientation toward obese individuals (Puhl and Heuer, 2009) and their subordinate status in the workplace, as evidenced by their lower salaries. However, little is known about obese people's implicit acceptance of their subordinate status.

In our experimental setting, subjects were asked to reveal “the amount of money they would like to request as compensation for their effort and for the information they have provided for fulfilling a questionnaire.” We expect that due to the self-weight bias, obese individuals would make on average lower monetary requests. An open-ended question was used (based on Greig, 2008), in order to reflect the salary negotiation process in a job-interview environment, where the job-candidate is asked to reveal his aspirations first. On top of the well documented wage discrimination against obese people, we suggest that a fraction of the wage gap across weight can be attributed to the lower initial salary requests (due to self-weigh bias) between obese and non-obese individuals, as these can serve as anchors in the negotiation process and influence subsequent offers and final agreements (Tversky and Kahneman, 1974; Ritov, 1996; Galinsky and Mussweiler, 2001).

In this study, using data of 270 subjects who were invited to claim money for filling in a questionnaire, we find robust evidence in favor of our self-weight bias hypothesis, namely that self-identified obese individuals claim a lower amount of money because they have implicitly accepted that they deserve less.

The common task for all participants was the completion of a 30-min (including instructions) questionnaire. Subjects were asked to self-report weight status and other appearance and personality characteristics together with other socioeconomic questions and a psychological test which was designed to distract subjects' attention from the real focus of this study. We use this self-identified weight status (*self-weight* henceforth) to categorize participants and test our primary hypothesis. Such self-reported measure has been also used before in Bosch-Domènech et al. (2014) and was found to be highly correlated with self-reported BMI. In a similar (to ours) experimental setting including self-reported questionnaires and monetary incentives, Brañas-Garza et al. (2016) found that self-reported BMI is not related to social preferences (altruism, fairness and trust). In a study closer to our self-weight bias hypothesis (Durso and Latner, 2008), internalized weight bias (measured by Weight Bias Internalization Scale) was found to be significantly correlated with antifat attitudes, lower self-esteem, body image concern, drive for thinness and measures of mood and eating disturbance. However, in the study by Puhl et al. (2007), internalized weight bias (measured by the degree to which participants believed stereotypes to be true or false) was not related to types or amount of stigma experiences reported, self-esteem, depression, or attitudes toward obese persons.

Additionally to *self-weight*, we have asked the 27 monitors who conducted the experiment to evaluate participants' weight (henceforth *monitors' weight*) using the same Likert scale. A replication of the analysis using *monitors' weight* instead of *self-weight* do not produce any significant result related to self-weight bias. Like in other studies on internalized weight bias (Puhl et al., 2007; Durso and Latner, 2008), using a self-reported measure of obesity is more relevant for approximating self-weight bias. As an additional test we compute the difference between the two measures (*self-weight* vs. *monitors'-weight*) to generate a new measure, the self-weight *overstatement*, which was found to be the key factor for the self-weight bias; *the excessive weight felt by the “self” but not reported by others (monitors) is a good predictor of lower money requests, not only for obese but also for non-obese individuals*.

In this study, we also attempt to shed light to the mixed findings in the literature related to the interaction between gender and weight bias. Starting from the gender literature, several studies (among others, Rosenbaum, 1984; Gerhart, 1990; Barron, 2003; Greig, 2008) have shown that women make significantly lower salary requests than men. However, when focusing on the obesity literature, the gender effect is ambiguous. While the meta-analysis by Roehling et al. (2008) showed that both overweight men and women were equally susceptible to weight discrimination, other earlier empirical studies have found that the obesity wage penalty is stronger for Baum and Ford (2004) and Averett and Korenman (1996) or only applies to females (Register and Williams, 1990; Pagan and Davila, 1997). Similarly, contradicting gender effects have been evidenced in the *internalization weight bias* literature with a study identifying a positive effect (Lillis et al., 2010) while others no association between females and weight bias (Puhl et al., 2007; Durso and Latner, 2008). In our study, we find only “weak” evidence for gender differences in self-weight bias, in the sense that the difference in money requests between self-identified obese and non-obese females are more significant than the respective differences between self-identified obese and non-obese males.

This paper adds to the literature in number of ways: First, we develop a genuine implicit proxy for experimentally eliciting the weight bias internalization. Second, we find that only self-reported measures of obesity are relevant to self-weight bias since they capture how people feel rather than how people really are or how they look to others. Third, we find that self-identified obese individuals experience larger self-weight bias (expressed by lower money requests). Finally, we find that the self-weight overstatement, which is the difference between self-reported and external interviewer's evaluation on subject's weight status, is the key factor behind individuals' self-weight bias.

After this introduction, the remainder of this study is organized as follows: the experimental methods are described in Section 2, while results are presented in Section 3. Section 4 concludes with a discussion of the results.

## 2. Materials and methods

We conducted an economic field experiment with 270 subjects from different socioeconomic backgrounds. Twenty seven monitors, aged between 20 and 60 years and from varying socioeconomic backgrounds were recruited to serve as monitors. All of them were students at the School of Social Work at the Universidad of Granada taking a module on *Economic Analysis of Social Work*. None of them had any previous experience with economic experiments.

### 2.1. Stage 1: monitors' training and preparations

Monitors were trained for a total of 6 h. Training included a general description of the experimental methodology with special reference to the experimental protocols of the present study. Additional instructions regarding the experiment were also given in detail. Each monitor was asked to independently recruit ten subjects to participate in an economic experiment within 1 week's time. The monitors had no information about the research focus of the study. By doing so it was ensured that subjects were not selected on the basis of any specific characteristic, thus avoiding any potential demand effect or sample bias.

The monitors were also told that they should aim for a balanced subject pool in terms of gender and employment status. This was done because we were interested in eliciting valuations from individuals who were in a workplace environment. After the first week, the monitors were asked to submit a list with the codified names (in order to assure anonymity) of the ten subjects they had recruited.

### 2.2. Stage 2: questionnaires and implementation

In the second stage, every monitor answered a questionnaire (*Q*_*m*_) describing each one of the 10 subjects she had recruited. The questionnaire consisted of three parts; Part 1: appearance and personality questions of the subjects, Part 2: Sally-Ann task (Wimmer and Perner, 1983), which was simply used as a distraction from the research focus, Part 3: Monitors described the nature of the relationship between herself and each one of her subjects.

After completing and submitting *Q*_*m*_ to the researchers, the monitors received 10 new questionnaires *Q*_*s*_ and 10 envelopes for each one of her subjects. These envelopes were delivered by them to their subjects for enclosing their private answers. Note that the first two parts of *Q*_*m*_ and *Q*_*s*_ were identical. The only difference is that the questions in *Q*_*m*_ were answered by each of the interviewer (describing the 10 participants) while the questions in *Q*_*s*_ were self-reported by each one of the 10 subjects (See Supplementary Materials for an English translation of the main parts of the *Q*_*s*_ questionnaire).

Since Part 2 of the questionnaires was only used to distract participants (and monitors) from the main goal of the research, we will focus here only on Part 1. It consisted of four questions about their appearance, namely obesity, beauty, height and manner of dress, and five questions about their personality characteristics, namely ambition, self-esteem, sociality, creativeness and benevolence. All these questions were ranked on a 7-level Likert scale. *Obesity* is used as an explanatory variable while *beauty*, *ambition*, and *self-esteem* are used as control variables. The remaining questions were not related to the experiment but were used to distract subjects (just like the Sally-Ann task in Part 2).

At the end of the *Q*_*s*_ questionnaire, in Part 3, participants were also asked regarding how much money they would like to receive for the task. Specifically, subjects were asked the following question: *How much money you would like to request as a compensation for the effort you made to fill out the questionnaire and for the information you provided us*. An alternative elicitation mode would be to ask subjects to select between, for instance, 0€, 5€, 10€, 15€, and 20€. However, this would anchor our participants' choices. In contrast, unrestricted question mode, avoid framing subjects elicitations. In fact, requesting for a very large amount was an option, which is of interest for the study.

It was also clarified that the money available for the research project was provided by the Spanish government and did not belong to either the monitors or the researchers. As the experiment took place in the field, subjects were also asked to give their names and home addresses for receiving the money that would be paid to them. Payments were realized 2 weeks later according to the following rule (unknown to the subjects and monitors ex-ante): Subjects who requested 10€ or more, were paid exactly 10€. All the rest received the exact amount of their request. Finally, subjects were asked whether they would be willing to participate in any other similar study and how much money the would presumably request for doing so.

### 2.3. Ethical concerns

All participants were assured that that their anonymity would always be preserved (in agreement with the Spanish Law 15/1999 for Personal Data Protection). Subjects were informed that no association will ever be made between subjects' real names, the corresponding codes and the final results. All experimental procedures were checked and approved by the Vice-Dean of Research of the School of Economics at the University of Granada, the institution coordinating the experiment.

## 3. Results

### 3.1. Data considerations and descriptive statistics

**Figure 1:**
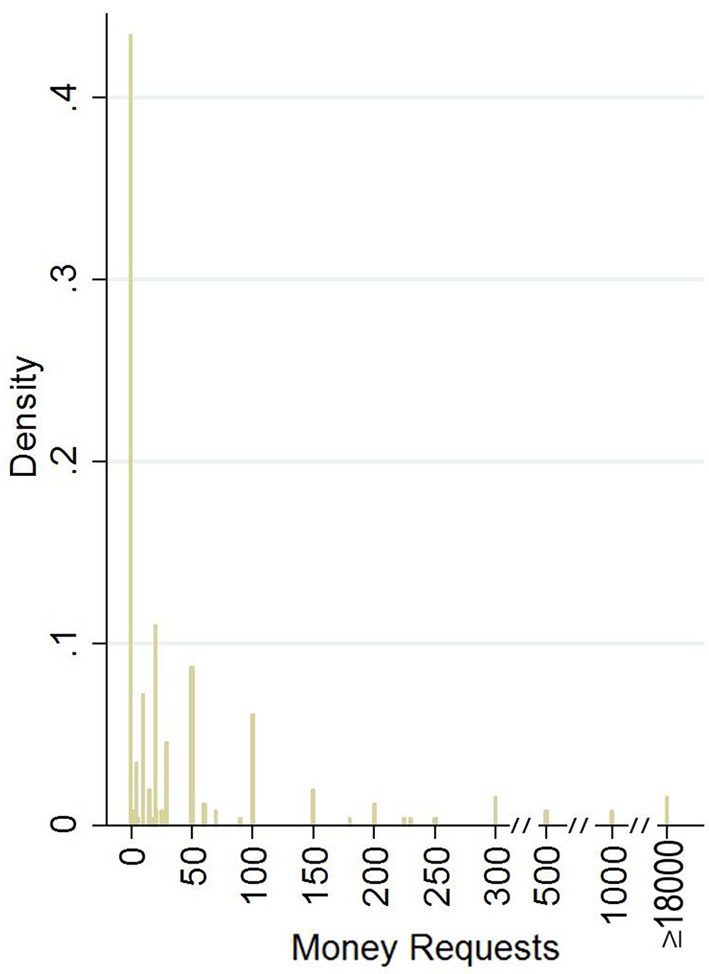
**Money requests histogram**. Distribution of subjects' money requests. The zero category includes also the 23 subjects who did not make any request (blank repsonse). X-axis value-intervals has been shortcuted for values more than 300. The open interval ≥ 18000 includes all remaining (4) extreme values.

Subjects' “money requests” (henceforth *requests*) is the main dependent variable under consideration. The empirical distribution of this variable has been found (Figure 1) not linear including many zeros (93 subjects requested 0€ and 23 gave blank answers), discontinuities, focal points (10€, 20€, 30€, 50€, 100€) and extreme values (4 values ≥ 18,000€, when standard deviation of *requests* is about 5830€). For this reason a 6-category variable (henceforth 6*cat_requests*, see Supplementary Table S1) with ordered values which are including at least one focal point of *requests* has been generated and will be analyzed in parallel with the original variable *requests*. Among others, such a transformation has the advantage of including all those extreme values which are eventually excluded as outliers from the regression analysis due to the distortion effect on the coefficients. These values are important for our analysis as they capture participants' intention to receive the highest possible stake.

Subjects were asked to fill in a questionnaire about their appearance and personality characteristics using 7-point Likert scales. Self-reported weight status (*self-weight*) was used as the main independent variable of this study. The original measure takes values from 1 (very thin) to 7 (very obese). However, for representation reasons which will become more obvious when describing the regression analysis, we separate “obese” (*self-weight* ≥ 5, henceforth *self-obese*) from “thin” (*self-weight* ≤ 3, henceforth *self-thin*) individuals. Self-reported beauty (1: very ugly to 7: very beautiful), self-esteem (1: no self-esteem at all, to 7: high self-esteem) and ambition (1: not ambitious at all to 7: very ambitious) are also included in the regressions analysis to control for possible confounding effects. The continuous variables *age* (and *age*^2^) and *wage* together with the dummy variable *female* were reported by the monitors and also used as controls variables. In the last part of the analysis, monitors' estimation of subjects' weight (*monitors' weight*) is also used for describing participants' weight status overstatement.

Table 1 summarizes the descriptive statistics for all these variables in their original form. The subject pool was comprised of 55% females and 35% university students. About 37% of the subjects were unemployed and 18% worked in a low-paid job (i.e., ≤ 850 €, corresponding to the lower quartile of our sample).

**Table 1:** **Descriptive statistics**.

**Variable**	***N***	**Mean**	**Median**	**Mode**	**Std. Dev**	**Min**	**Max**
*self-weight*	269	4.18	4	4	1.05	1	7
*beauty*	269	4.79	5	5	0.97	1	7
*ambition*	269	4.52	5	5	1.34	1	7
*self-est*.	269	4.49	5	5	1.48	1	7
*monitors' weight*	270	3.65	4	4	1.43	1	7
*female*	270	0.55	1	1	0.50	0	1
*age*	270	29.33	25	24	9.47	18	65
*wage*	171	1316	1200	1500	848	100	7000

*One subject did not answer the Q_s_ questionnaire at all, reducing our sample to 269 self-reported observations. Monitors reported Q_m_ questionnaires for all 270 subjects. The variable wage refers to the monthly salary of the 171 subjects who currently have a job*.

It is interesting to see that the mean, the median and the mode of the self-reported variables *beauty*, *ambition* and *self-esteem* are much higher than expected (i.e., 4, assuming a normal distribution). However, with regards to *obesity* the mean value approaches the expected one, while the mode and the median are exactly 4. This is probably due to the fact that weight status is an obvious appearance characteristic, leaving little space for subjective mis-estimations.

### 3.2. Self-weight bias

In this section, we will start our analysis with a graphical representation of the relation between the variables *requests* and 6*cat_requests* with *self-weight*. As a second step of analysis, we will conduct some preliminary non-parametric tests which will provide a first confirmation of our self-weight bias hypothesis. Finally, by performing linear (OLS) and non-linear (Tobit, Probit) regression analysis (Table 2), we will control for potential confounding factors and also account for some of the specific characteristics of our data (intra-group correlation, outliers, non-linearity of the dependent variable, etc.).

**Table 2:** **Regressions on money requests**.

	**OLS (1) Requests**	**Tobit (2) Requests**	**Tobit (3) Requests**	**o-Probit (4) 6cat_requests**	**Probit (5) 2cat_requests**
*self-obese*	−30.514^***^	−61.889^***^	−24.316^***^	−0.422^***^	−0.340^**^
	(10.882)	(23.097)	(7.477)	(0.124)	(0.150)
*self-thin*	−8.844	−29.150	−10.464	−0.230	−0.348
	(15.767)	(27.021)	(11.595)	(0.212)	(0.253)
*female*	−18.545	−17.797	0.385	0.000	0.056
	(14.359)	(22.687)	(10.081)	(0.170)	(0.200)
*beauty*	8.558	14.771	4.390	0.074	0.110
	(11.740)	(15.863)	(5.043)	(0.078)	(0.082)
*age*	−13.776^**^	−24.596^**^	−7.774^**^	−0.128^**^	−0.133^**^
	(6.045)	(9.825)	(3.347)	(0.057)	(0.060)
*age*^2^	0.167^**^	0.282^**^	0.081^*^	0.001^*^	0.001^*^
	(0.080)	(0.130)	(0.044)	(0.001)	(0.001)
*wage*	0.018	0.012	0.001	−0.000	−0.000^*^
	(0.013)	(0.019)	(0.007)	(0.000)	(0.000)
*ambition*	9.297^**^	15.226^*^	6.123^*^	0.098	0.060
	(3.835)	(8.029)	(3.620)	(0.063)	(0.073)
*self-est*	−5.012	−2.859	1.317	0.027	0.042
	(7.095)	(10.523)	(3.581)	(0.060)	(0.066)
*cons*	233.673^**^	333.905^**^	112.705^**^		2.059^*^
	(109.691)	(153.168)	(53.569)		(1.107)
**N**	265	265	269	269	269
*R*^2^ (pseudo)	0.092	0.017	0.023	0.047	0.109
*Prob* > *F*/χ^2^	0.0494	0.00958	0.00115	0.00000424	0.0000797

*Standard errors (adjusted for 27 clusters in monitors) of parameters estimates in parentheses*.

* *p < 0.10*,

** *p < 0.05*,

*** p < 0.01. (1) and (2): Four observations are excluded as outliers (> 3^*^s.d.) (2) and (3): 154 left-censored observations at requests = 0. (3): 24 right-censored observations at requests > 100. (4) 6cat_requests: six ordered values around the focal points (0, 10, 20, 30, 50, 100). Cut points are omitted. (5) 2cat_requests: dichotomous variable (=1 if requests > 0, 0 otherwise).

**Figure 2:**
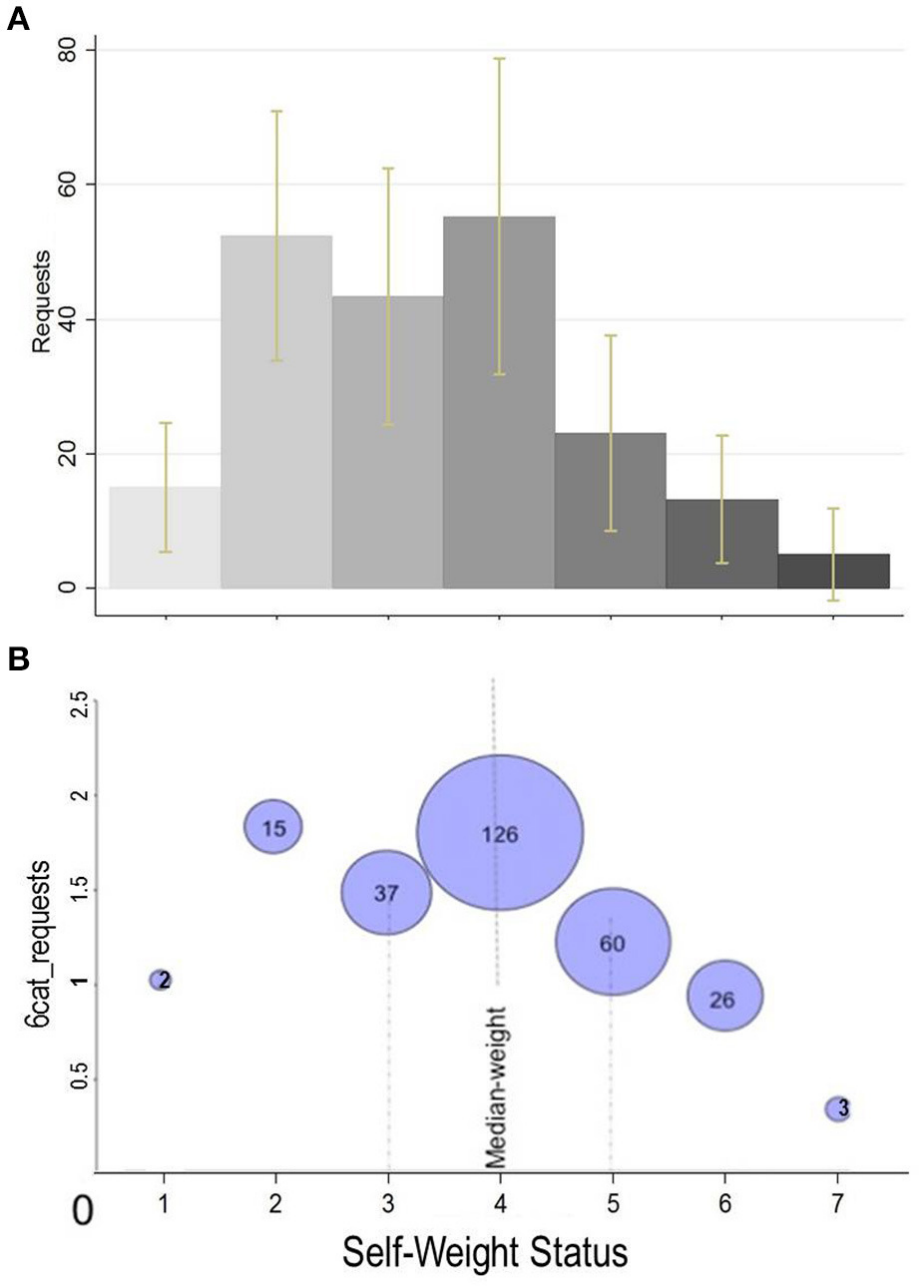
**Mean ***requests*** and ***6cat_requests*** by self-reported obesity level**. **(A)** *Requests* refer to the original variable (excluding outliers for *requests* ≥ 18000, *n* = 265). Yellow bars: 95% confidence interval. **(B)** *6cat_requests* is a 6-value ordinal transformation of the original variable (*n* = 269). The size of the bubble is proportional to the number of individuals in that category.

Figure 2 shows the means of (Figure 2A) *requests* (including the 95% confidence intervals) and (Figure 2B) 6*cat_requests* by the 7 different levels of *self-weight*. The size of the bubble in (Figure 2B) is proportional to the number of people belonging to each level of *self-weight*. Note that the *self-weigh* value 4 (horizontal axis) corresponds to those subjects who consider themselves neither *thin* nor *obese*.

At high values (5–7) of *self-weight*, a negative trend is already visible from the figure. This is also supported by Mann–Whitney (henceforth M-W) non-parametric tests; for the variable *requests* [after having dropped out the four extreme values (i.e., ≥ 3^*^std, *n* = 265)], we found that individuals with *self-weight* of levels 5 (*sw*5) or 6 (*sw*6) are requesting significantly less (at 5%) as compared to participants with *self-weight* level 4 (*sw*4). [M-W: (*sw*4 vs. *sw*5; *z* = 2.49, *p* = 0.013), (*sw*4 vs. *sw*6: *z* = 2.09, *p* = 0.037)]. The same result is replicated when the variable 6*cat_requests* (*n* = 269) is used [M-W: (*sw*4 vs. *sw*5; *z* = 2.28, *p* = 0.022), (*sw*4 vs. *sw*6: *z* = 2.24, *p* = 0.025)]. We do not run any non-parametric test for *sw*7 as too few observations (*n* = 3) are included in this category.

On the other hand, there is no clear pattern for the average requests among self-identified thin (values 1–3) people. This is probably due to the fact that ranking of *self-weight* lower values are not really as straightforward as other variables. For instance, beauty is clearly monotonic in its self-ranking (e.g., a person of *beauty* = 6 is always considered better off than a person of *beauty* = 2). In contrast, really low values in the weight scale might be perceived equally bad as really high values (somebody can think of him/her self as too thin). It is therefore plausible to split the *self-weight* variable into two different dummies: *self-obese* taking the value of 1 if *self-weight* ≥ 5 and 0 otherwise, and *self-thin* taking the value of 1 if *self-weight* ≤ 3 and 0 otherwise.

In the following regression analysis, money requests are regressed over these two dummy variables for facilitating presentation (main results are also replicated in Supplementary Table S1 when the original 7-point *self-weight* variable or *ob*3 (*ob*3 = *requests* if *requests* ≥ 5, 0 otherwise) are used in the regression instead of the two dummies). The original 7-point measures of *beauty*, *ambition*, and *self-esteem*, the continuous measures *age* (and *age*^2^) and *wage* and the dummy variable *female* are also used in the regressions as control variables. Coefficients and standard errors (in parentheses) of all these regressors are presented in Table 2. We also account for the potential monitors' influence on their subjects decisions by allowing for intra-group correlation and relax the usual requirement that the observations be independent (i.e., 27 clusters for different monitors). Although monitors were specifically instructed not to influence subjects' answers, we cannot ignore that subjects may have been recruited from the monitor's proximate environment.

As robustness check, in Table 2, our dependent variable—money requests—is grouped and regressed in five different ways: In (1)–(3) the original variable *requests* is used. In (1) and (2) after the four extreme values (≥ 3^*^std) exclusion (*n* = 265), OLS and Tobit (left-censored at *requests* = 0, *N*_*lc*_ = 154) regressions are used respectively. In (3) all values are included (*n* = 269) in the Tobit regression but eventually censored out (*N*_*rc*_ = 24 at *requests* > 100 and *N*_*lc*_ = 154 at *requests* = 0). In (4) we use an ordered-Probit regression on the six-ordered variable 6*cat_requests* mentioned earlier. Finally, in (5) the dichotomous variable 2*cat_requests* (=1 if requests > 0, 0 otherwise) is regressed with a Probit model to answer the question who is more prone to request a positive amount of money.

Censoring from below in (2) and (3) seems quite plausible as zero appears as the natural lower bound, although some participants would be theoretically willing even to give money instead of receiving (alternatively, there were people willing to fill in even larger questionnaires without any compensation). This is probably the case of the 99 participants who requested 0€ not only in our main question but also when asked “*For which amount of money will you be willing to participate in a future study?”* (see Supplementary Material). Censoring from above in (3) has a post-experimental corrective scope. The open-ended question used for eliciting money-requests (Greig, 2008) has the advantage of excluding any anchoring effects (Tversky and Kahneman, 1974) but also the disadvantage of allowing really high requests which later complicate the analysis of our data. Assuming that the intention of those people requesting high stakes was simply to demonstrate that they want the highest possible payment (i.e., a person requesting 15,000€ or 500€ has the same intention with a person requesting 100€), we can also censor the data from the right. As mentioned earlier, this is also the logic behind the highest category in 6*cat_requests* which also includes the 4 outliers.

All regressions confirm the negative association between the dependent variable and *self-obese* at 1% significance level in (1)–(4) and at 5% in (5). In OLS regression (1) where coefficients have a straightforward interpretation the result is striking: *self-obese* individuals' requests are at least 30€ less than the corresponding requests of the median group with *self-weight* = *4* (henceforth *non-obese*). Censoring out from below the 154 zero requests in (2), the linear effect of *self-obese* on the uncensored latent variable is doubled as *self-obese* individuals request almost 62€ less than the control group. When we additionally censor *requests* from above in (3) for high values (> 100), the linear effect of *self-obese* on the uncensored latent variable is similar to the OLS result: *self-obese* individuals request 24€ less than their non-obese counterparts. Interestingly, this result remains significant (coefficient = 16.93, *p*_*value*_ = 0.002) even when data is censored from above at a lower level (*requests* > 15€).

**Figure 3:**
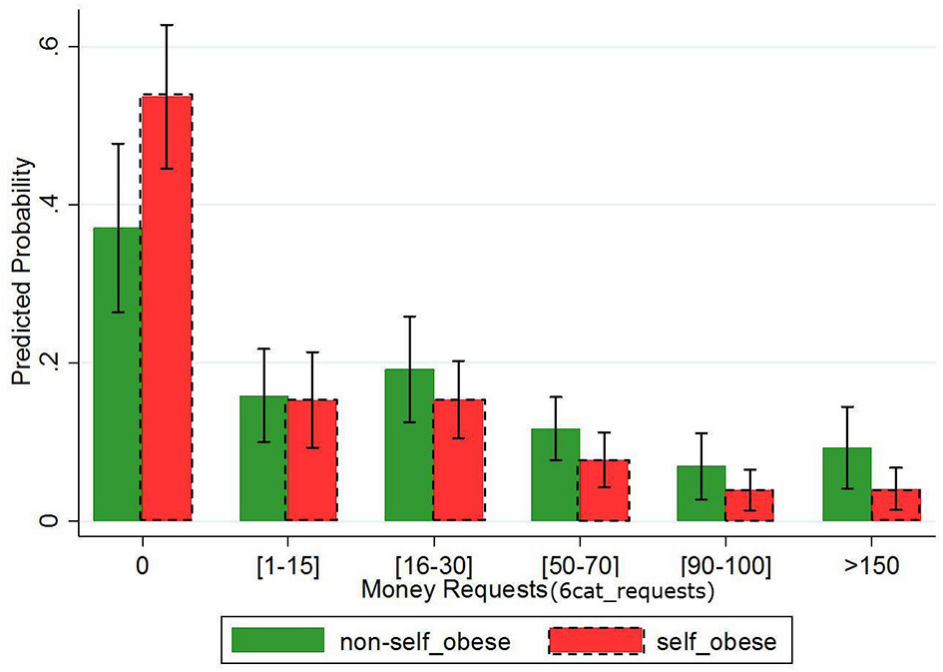
**Predicted probabilities for each category of ***6cat_requests*** by ***self-obese*****. Ordered Probit predictions of *6cat_requests* calculated for *self-obese* (red dash-framed bars) and *non-obese* (green bars) separately after having fixed all other predictors at their mean value.

In (4) the effect of self-obese on 6*cat_requests* is negative and highly significant (*p*_*value*_ < 0.001). In Figure 3, we present the predicted [after having performed (4)] probabilities to belong to each one of the 6*cat_requests* categories for *self-obese* and *non-obese* individuals separately, when all other predictors are fixed at their mean value. The probability of *self-obese* individual requesting 0 is 45% (i.e., [*pr*(0|**self-obese)**/*pr*(0|**non-obese**)]−1) higher than the corresponding probability of a *non-obese* individual. At the same time, *non-obese* has 130%, 77%, and 51% more chance to fall in the category 5 (i.e., *requests* > 150), 4 (i.e., *requests* ∈ [90−100]) and 3 (i.e., *requests* ∈ [70−90]), than their *self-obese* counterparts, respectively. *Self-obese* individuals' preference over zero requests and the one of *non-obese* for positive requests is exactly captured by the respective coefficient in the Probit model (5). These results are summarized as follows:

**Result 1:** *In comparison to non-obese, self-identified obese individuals request significantly less money and are more prone not to request any money at all*.

In other words, the self-weight bias hypothesis that obese people have internalized the negative attitudes toward themselves and behave in a different way than non-obese people by claiming less or nothing is confirmed even after controlling statistically for a series of potential confounding factors.

Interestingly enough, no clear cut results are obtained when we study the self-reported measure of *self-thin* and *beauty* with any of our dependent variables and regressions. More specifically, the variable *beauty* does not capture any effect even in the absence of *self-obese* and *self-thin* variables from the models (not reported here). Moreover, the variable *self-esteem* was not found significant in any of the regressions, justifying the ambiguous role of self-esteem in Social Identity Theory. The fact that someone belongs to a “high-status” group (thin or normal-weight) may increase self-esteem but on the other hand the reason why someone is seeking to join in a group could be related either to low or to high self-esteem. Regarding the rest of the control variables, *age* is associated (negatively) with the dependent variables in a significance level lower than 5% in all regressions while ambition seems to have a positive effect only in OLS and Tobit regressions.

**Figure 4:**
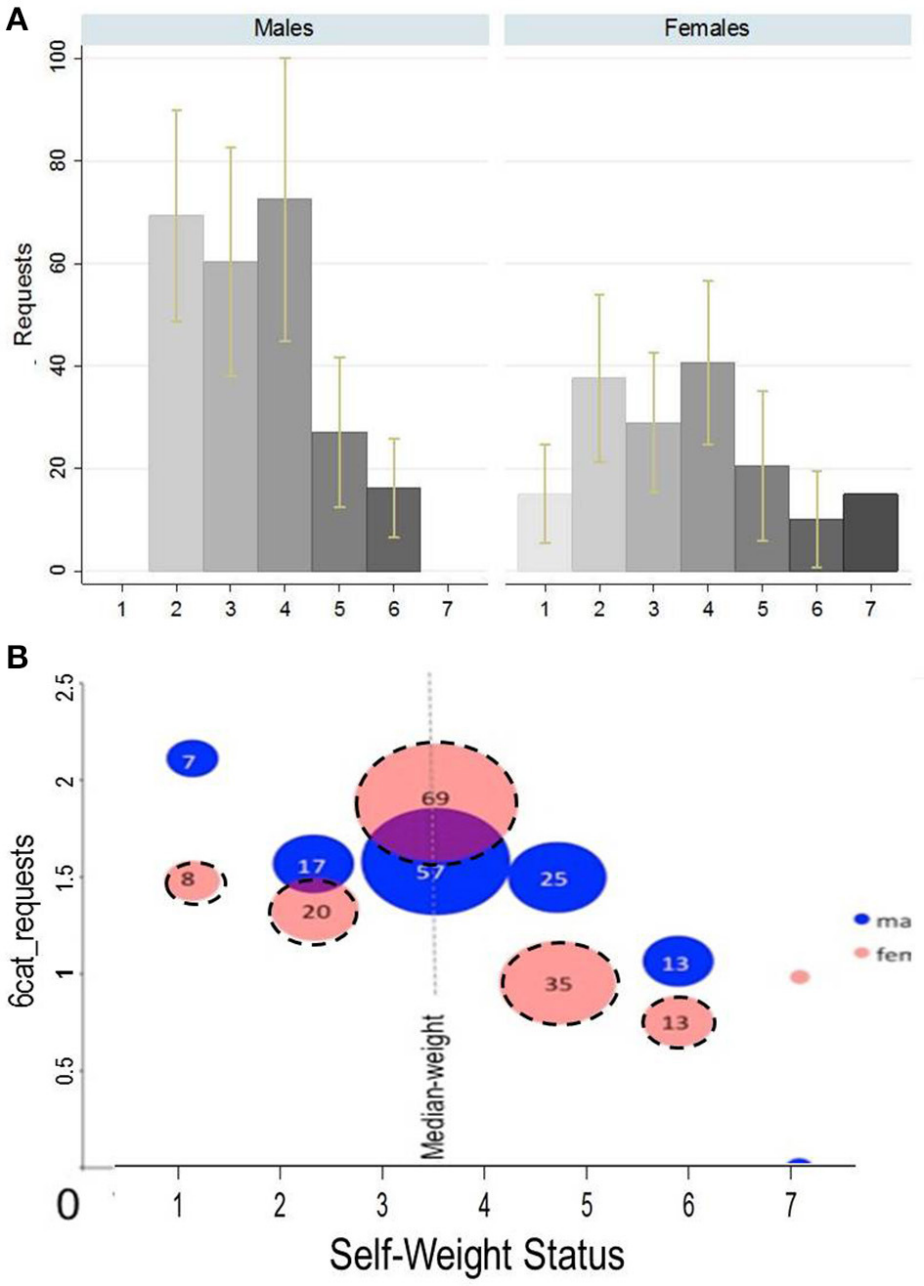
**Mean ***requests*** and 6***cat_requests*** by ***obesity*** level and gender**. **(A)** *Requests* refer to the original variable (outliers are excluded). Yellow bars: 95% confidence interval. **(B)** *6cat_requests* is a 6-categorical ordinal variable. Red dash-framed bubbles correspond to females and blue ones refer to males. The size of the bubble is proportional to the number of individuals in that category.

Now we turn our attention to gender effects. Figure 4 illustrates the average *requests* or 6*cat_requests* by *self-weight* level and gender. In Figure 4A results are not really representative as the average requests in some obese categories are influenced by some extreme values. This problem is eliminated with the 6*cat_requests* transformation illustrated in Figure 4B. Although we have not found the variable *female* significant in any of the earlier regressions, Figure 4B shows that the negative trend between 6*cat_requests* and *self-obese* (i.e., *self-weight* ≥ 5) is stronger in the female subsample.

However, the interaction between gender and *self-obese* (or *self-weight* or *self-ob3*, see Supplementary Table S2) is not significant in any of the OLS regressions. In absence of a direct calculation for standard errors for the interaction term (Ai and Norton, 2003) in Probit models, we repeat Probit analysis in Supplementary Table S3 for the female and male subsamples separately. Only *self-obese* females request significantly less money (both with 6*cat_requests* and *2cat_requests*, at 1 and 5% significance level respectively) than non-obese females. In the male sample, although the negative sign holds, the variable is not significant. Result 2 is summarized as follows:

**Result 2:** *The evidence for gender difference on self-weight bias is weakly supported: The negative association between self-obese and the categorical variables* 6*cat_requests or* 2*cat_requests remains significant but only for the female subsample*.

However, we do recognize that this result is partially affected by the loss of statistical power due to the restricted number of observations in the two subsamples.

### 3.3. Monitors' evaluations and the self-weight overstatement

As no traditional objective measure of obesity (actual weight, BMI, etc.) was included in our study, it is important to check the discrepancy between *self-weight*, and monitor's reports on subjects' weight status (*mon_rep_weight*). Interestingly, the percentage of individuals who overstate their weight status in the *self-obese* category (62%) is significantly higher than those who understate or accurately state it in both *self-thin* (42%) and *self-normal* (44%) categories (MW: *p* = 0.028 and *p* = 0.010 respectively, see also Supplementary Figure 2). We repeat OLS regressions using the monitor reported obesity variables and we find no significant effect [see Table 3 for *mon_rep_obese* and also Supplementary Table S4 using *mon_rep_weight* and *mon_rep_ob*3 (= *mon_rep_weight* if *mon_rep_weight* ≥ 5, 0 otherwise) as main regressors]. This indicates that self-weigh bias is only affected by subjective (self-reported) measures of obesity and not by others' evaluations. This result is summarized as follows:

**Result 3:** *The main determinant of the self-weight bias is the self-perceived own-weight status. Others' evaluations on subjects' weight status do not affect the self-weight bias*.

**Table 3:** **OLS on requests with monitors' reports**.

	**(6) Requests**	**(7) Requests**	**(8) Requests**
*mon_rep_obese*	−24.397	−31.806	−32.778
	(16.748)	(19.587)	(22.428)
*weight_overstate*		−28.768^**^	−29.939^*^
		(12.080)	(15.424)
mrobese^*^overstate			6.433
			(23.616)
*age*	−14.185^*^	−13.749^*^	−13.715^*^
	(7.312)	(6.988)	(7.023)
*age*^2^	0.168^*^	0.166^*^	0.165^*^
	(0.096)	(0.092)	(0.093)
*cons*	297.032^**^	292.520^**^	292.693^**^
	(138.123)	(133.288)	(133.743)
**N**	264	264	264
*R*^2^	0.066	0.076	0.076
*Prob* > *F*	0.448	0.0933	0.0532

*Standard errors (adjusted for 27 clusters in monitors) of parameters estimates in parentheses*.

* *p < 0.10*,

** p < 0.05. Four observations are excluded as outliers (> 3^*^s.d.) and 1 as a missing value. All variables starting with mon_rep_ refer to monitors' reports. weight_overstate takes the value 1 if self_weight-mon_rep_weight > 0, and 0 otherwise. Controls based on monitors' reports mon_rep_thin, mon_rep_beauty, female, wage, mon_rep_ambition, mon_rep_self_est are used but omitted as no significant.

In regressions (7) and (8), we combine self-reported and monitor information in the same regressions by including the variable *weight_overstate* and its interaction with *mon_rep_obese*, mrobese^*^overstate. The variable *weight_overstate* is a dummy variable which takes the value 1 if *self_weight*−**mon_rep_weight** > 0, and 0 otherwise. In other words *weight_overstate* captures all those subjects who perceive themselves more obese than their respective external evaluator. We see that *weight_overstate* is significant in (7) while the *mon_rep_obese* remains insignificant. This means that self-weight bias (as approximated by money *requests*) is not associated with objective obesity (as evaluated by monitors) but only with the excessive weight (over monitors' estimation) which was self-reported by subjects. In particular, weight-status overstatement reduces *requests* by almost 29€, counterbalancing almost all the effect which was previously captured in (1) by the *self-obese* variable.

**Figure 5:**
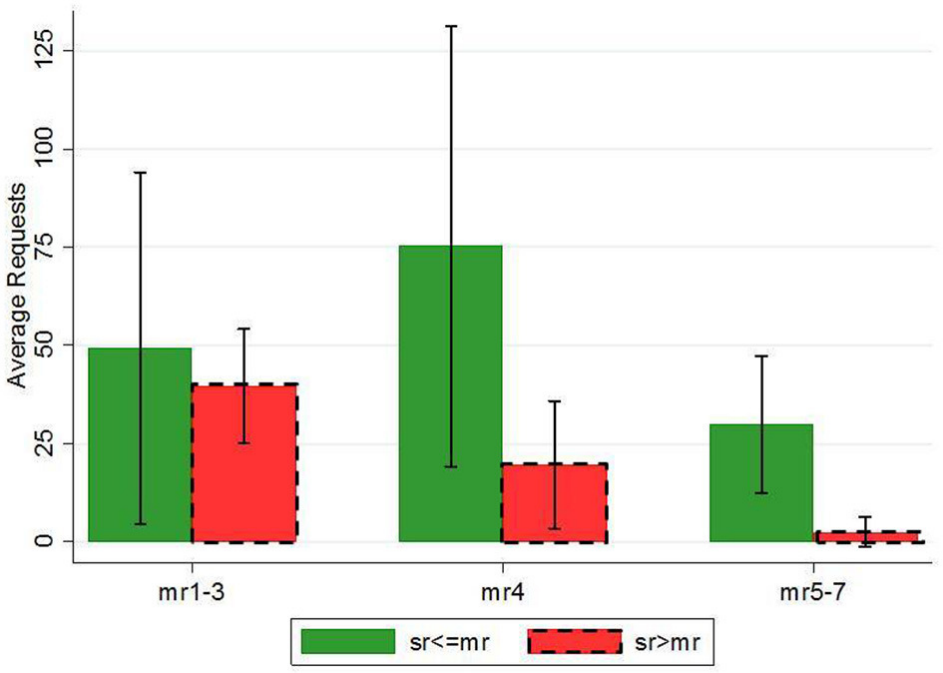
**Requests by ***self-weight*** (≥ 4) and weight overstatement**. *Self-weight* is considered overstated when self-report>monitor-report (red dash-framed bars). Error bars represent 95% confidence intervals. *Self-weight* = *7* has been eliminated due to minor number of observations in the respective categories.

More importantly, the fact that in (8) the interaction term *mrobese* ^*^ *overstate* is not significant shows that the negative effect of self-weight overstatement (henceforth *overstate*) applies to all weight-status levels and not only to *mon_rep_obese* individuals. Figure 5 illustrates exactly this last result (as robustness test see also Supplementary Table S4 including the original variable *mon_rep_weight* or *mon_rep_ob*3). Although the effect is negative in all obesity levels (justifying the non-significance of *overmrobese*), the differences in *requests* between *overstate* and *non-overstate* individuals is significant (MW: *z* = 1.852, *p* = 0.064) only in *mon_rep_obese* (*mon_rep_weight* ≥ 5) category.

**Result 4:** *The excessive weight felt by the “self” but not reported by the external evaluators determines the self-weight bias not only for obese but also for non-obese individuals*.

## 4. Discussion

In this study, we have tested for the existence of internalized weight bias in people who self-report high weight status. Following experimental economics methodology, we have developed an implicit measure of self-weight bias by giving the same monetary incentives to both obese and non-obese persons. The experimental setting was actually simulating a salary negotiation environment in which participants were asked to state their money request for performing the same simple task. We found that self-identified obese individuals made significantly lower monetary requests as compared to non-obese. We therefore suggest that part of the obesity wage-gap is explained by obese individuals' lower reservation wages. We moreover have elicited monitors' estimations on subjects' weight status and used this information for comparison with subject's self-reports. We find that those individuals who overstate their weight status as compared to monitors' evaluation were those who were actually experiencing the self-weight bias presumably due to “*false consciousness*.” More importantly, we find that the self-weight bias is not only experienced by individuals who were characterized (by their monitors) as non-obese but also by those who were characterized as non-obese.

However, a different interpretation of this last result is possible, assuming that individuals' weight status is self-reported correctly but underestimated by monitors. Monitors' kindness or even sympathetic feelings especially toward obese individuals may give an alternative explanation to the self-weight bias. Monitors are more conservative to their evaluations in an attempt to be more gentle toward the sensitive (with the obesity issue) obese individuals. Regardless of the reference point and the consequent interpretation, the robust result remains the same: self-overstated or monitors' under-evaluated individuals are experiencing a self-weight bias.

To our great surprise self-esteem did not play any role in our study. Although socio-psychologists have highlighted the negative relationship between self esteem and obesity (French et al., 1995; Miller and Downey, 1999; Hesketh et al., 2004; Carr and Friedman, 2005; Biro et al., 2006), we find no association between our self-reported weight status and self-esteem variables. More importantly, self-esteem never appears significant in any of the regressions we have performed. One argument is that people who feel closely attached to an in-group are those with low self-esteem (see Baumeister and Leary, 1995) who expect to be benefited from the affiliation (Klaczynski et al., 2004). Particularly when a group has a high social standing (e.g., “thin” women), individuals with low self-esteem should seek membership benefits more often and should identify more closely with the in-group's values than high self-esteem individuals (Bigler et al., 1997). On the other hand, the fact that someone belongs to a group may increase self-esteem due to solidarity feelings. The interaction between self-esteem and obesity becomes even more complicated when referred to low social standing groups (e.g., “obese” individuals) in which membership is not really desired.

Generally speaking, our findings are in accordance with the concept of *false consciousness*, extensively used by the System Justification Theory (Jost and Banaji, 1994). False anti-fat attitudes and stereotypes have been internalized by obese people leading to in-group devaluation and differential behavior. Along the same lines, Self-Fulfilling Prophecy Theory (Merton, 1948) would predict that obese people eventually shape their behavior in an expectancy-consistent manner, which justifies non-obese individuals' false general beliefs and differential treatment toward obese people.

We claim that our standardized experimental setting creates the appropriate conditions for eliciting self-weight bias. The selection of a minor task to be performed minimized the opportunity cost discrepancies across individuals with different characteristics and skills (i.e., the task was equally difficult for all participants irrespectively of their weight status). At the same time the standardized monetary incentive given to participants have created equal opportunities for all of them. Thus, we have accurately measured participants' reactions in our stimulus expressed in money requests. After controlling for other theoretically-based confounding factors, we have isolated the effect of obesity and estimated the self-weight bias.

Due to these controlled experimental conditions, we suggest that our findings can be extrapolated to other fields like in the labor market. Without underestimating the importance of actual wage discrimination against obese people, we offer a complementary explanation to the wage gap across weight; the intrinsic tendency of obese people to claim less may result in lower salaries. We therefore conclude that discrimination in the working environment expressed by lower wages is exacerbated (rather than generated) by self-weight bias as obese people start their negotiation from an inauspicious initial position.

Such a generalization of course has its limitations. As with the vast body of experimental studies, standard criticisms of the representativeness of our subject pool apply. Furthermore, monitors' influence on subject answers could only be controlled statistically. Another important caveat is that we model a one-shot interaction between subjects and monitors while in real life the salary negotiation process may last for longer, leaving time for both employers and candidates to readjust their strategies.

## Author contributions

The work is a product of the intellectual environment of both authors; and that both authors have contributed in various degrees to the analytical methods used, to the research concept, and to the experiment design.

## Funding

Financial support was received from grants by MCI (ECO2013-44879-R), the Regional Government of Andalusia (P12-SEJ-1436) and the European Commission.

### Conflict of interest statement

The authors declare that the research was conducted in the absence of any commercial or financial relationships that could be construed as a potential conflict of interest.
