# Supplementary material for: Self-identified Obese People Request Less Money: A Field Experiment
Source: Front Psychol. 2016 Sep 23;7:1454. doi: 10.3389/fpsyg.2016.01454 (PMC5033972; doi:10.3389/fpsyg.2016.01454)
Supplement: Supplementary file 1 [file Presentation1.PDF]

Supplementary Material:

Self-identified obese people request less money.

Antonios Proestakis* and Pablo Brañas-Garza

*Correspondence:
Author Name: Antonios Proestakis
antonios.proestakis@ec.europa.eu

1 SUPPLEMENTARY TABLES AND FIGURES

Supplamentary Table S1

Table S1

Dependent Variable: *6cat_requests*

label	0	1	2	3	4	5
requests (€)	0	$1 - 15$	$16 - 30$	$50 - 70$	$90 - 100$	≥ 150
n	116	39	46	28	17	24

o6-req is a 6-category variable with ordered values around the focal points (10, 20, 50, 100) of the original variable *requests*. The highest category ≥ 150 also includes the four extreme values ($\geq$ 18000€).

Supplamentary Table S2

Table S2: OLS with *self-obese*, *self-weight*, *ob3* and Gender Interactions.

	(1)	(2)	(3)	(4)	(5)
	requests	*requests*	*requests*	*requests*	*requests*
self-obese[a]	-44.951*				
	(21.883)				
obfem	26.059				
	(23.483)				
self-weight[b]		-9.029**	-13.559**		
		(3.972)	(6.176)		
weightfem			8.206		
			(7.991)		
self-ob3[c]				-19.299***	-23.607**
				(6.703)	(10.574)
ob3fem					8.668
					(10.528)
self-thin[d]	-8.769			-7.363	-7.159
	(15.604)			(15.179)	(14.989)
female	-26.510	-19.625	-53.731	-19.775	-23.483
	(19.273)	(14.259)	(39.475)	(14.611)	(17.430)
beauty	8.594	9.583	9.545	8.412	8.399
	(11.622)	(12.126)	(12.100)	(11.915)	(11.903)
age	-13.619**	-14.103**	-13.873**	-13.804**	-13.666**
	(6.028)	(6.392)	(6.363)	(6.135)	(6.082)
age^2	0.164**	0.171*	0.168*	0.166**	0.165*
	(0.079)	(0.084)	(0.084)	(0.081)	(0.080)
wage	0.018	0.018	0.018	0.018	0.018
	(0.013)	(0.013)	(0.013)	(0.013)	(0.013)
ambition	9.175**	8.959**	8.906**	8.850**	8.718**
	(3.690)	(3.787)	(3.750)	(3.846)	(3.738)
self-est	-4.393	-4.959	-4.796	-5.034	-4.676
	(6.922)	(7.126)	(7.063)	(7.067)	(6.986)
cons	233.132**	261.584**	276.256**	236.574**	235.199**
	(110.370)	(109.997)	(117.187)	(112.017)	(111.588)
N	265	265	265	265	265
R^2	0.095	0.084	0.085	0.091	0.092
$Prob > F$	0.0538	0.0659	0.0849	0.0538	0.0587

Note. Dependent Variable: *requests* (continuous). Independent Variables: b:1 (very thin) to 7 (very obese); a:1 if $b\geq$ 5, 0 otherwise; d:1 if $b\leq$ 3, 0 otherwise; c=b if $b\geq$ 5, 0 otherwise; *obfem*, *wightfem* and *ob3fem*: interactions of the dummy variable *female* and *a, b, c* respectively; *beauty*:1 (very ugly) to 7 (very beautiful); self-esteem: 1:(no self-esteem at all) to 7 (high self-esteem); ambition: 1(not ambitious at all) to 7(very ambitious); age, age^2 and $wage$ continuous variables. Standard errors (adjusted for 27 clusters in interviewers) of parameters estimates in parentheses.$^*p < 0.10$, $^{**}p < 0.05$,$^{***}p < 0.01$. Four observations are excluded as outliers (>3*s.d.).

Supplamentary Table S3

Table S3: Probit regressions on *6cat_requets* & *2cat_requets* by Gender

	6cat_requets		2cat_requets	
	females	males	females	males
	(4f)	(4m)	(5f)	(5m)
self-obese	-0.685***	-0.164	-0.625**	-0.111
	(0.208)	(0.193)	(0.266)	(0.225)
self-thin	-0.415	-0.059	-0.566*	-0.192
	(0.316)	(0.274)	(0.341)	(0.360)
female	0.000	0.000	0.000	0.000
	(.)	(.)	(.)	(.)
beauty	0.068	0.121	0.089	0.241*
	(0.102)	(0.132)	(0.105)	(0.136)
age	-0.077	-0.196**	-0.062	-0.235***
	(0.064)	(0.077)	(0.074)	(0.080)
age^2	0.001	0.002**	0.001	0.003**
	(0.001)	(0.001)	(0.001)	(0.001)
wage	-0.000	0.000	-0.000	-0.000
	(0.000)	(0.000)	(0.000)	(0.000)
ambition	0.094	0.090	0.081	-0.036
	(0.082)	(0.107)	(0.098)	(0.123)
self-est	-0.017	0.050	-0.072	0.196*
	(0.083)	(0.099)	(0.081)	(0.116)
cons			1.429	2.951*
			(1.409)	(1.665)
N	148	121	148	121
pseudo R^2	0.046	0.063	0.086	0.197
$Prob > \chi^2$	0.0000230	0.00482	0.00557	0.000149

Notes. Dependent Variables: In (4), *o6-req*: six ordered values around the focal points (0, 10, 20, 50, 100) of *requests* (Table S1). Cut points are omitted. In (5), *d-req*: 1 if *requests*$>$ 0, 0 otherwise. Independent Variables: *self-obese*:1 if *self-weight*$\geq$ 5, 0 otherwise; *self-thin*:1 if *self-weight*$\leq$ 3, 0 otherwise; *female*: dummy variable for females; *beauty*:1 (very ugly) to 7 (very beautiful); self-esteem: 1:(no self-esteem at all) to 7 (high self-esteem); ambition: 1(not ambitious at all) to 7(very ambitious); age, age^2 and $wage$ continuous variables. Standard errors (adjusted for 27 clusters in interviewers) of parameters estimates in parentheses.$^*p < 0.10$, $^{**}p < 0.05$,$^{***}p < 0.01$.

Supplamentary Table S4

Table S4: OLS with Monitors Evaluations and Overstatement Interactions.

	(1) requests	(2) requests	(3) requests	(4) requests	(5) requests	(6) requests
	Main regressor $mr - weight$			Main regressor $mr - ob3$		
mon_rep_weight[a]/*ob3*[b]	0.545	-6.483	-8.655	0.306	-3.711*	-4.275
	(4.232)	(4.727)	(8.274)	(2.034)	(1.927)	(3.278)
weight_overstate		-32.259**	-46.408		-31.623**	-35.998
		(13.992)	(49.505)		(13.590)	(28.701)
*weight/ob3*overstate*			3.777			1.299
			(10.521)			(5.089)
mon_rep_beauty	4.710	5.781	5.547	4.716	5.545	5.476
	(6.323)	(6.624)	(6.421)	(6.227)	(6.509)	(6.424)
female	-22.028	-23.944*	-24.149*	-22.154	-22.445	-22.569
	(13.067)	(13.653)	(13.903)	(13.289)	(13.520)	(13.714)
age	-13.706*	-13.307*	-13.264*	-13.713*	-13.246*	-13.256*
	(7.046)	(6.733)	(6.796)	(7.016)	(6.656)	(6.694)
age^2	0.160*	0.158*	0.158*	0.160*	0.158*	0.158*
	(0.093)	(0.090)	(0.091)	(0.093)	(0.089)	(0.089)
wage	0.018	0.016	0.016	0.018	0.016	0.016
	(0.015)	(0.014)	(0.014)	(0.015)	(0.015)	(0.015)
mon_rep_ambition	-0.127	-0.443	-0.492	-0.128	-0.411	-0.471
	(5.973)	(5.952)	(5.976)	(5.888)	(5.951)	(5.939)
mon_rep_self-est	-2.128	-1.234	-1.146	-2.127	-1.212	-1.117
	(7.633)	(7.293)	(7.164)	(7.643)	(7.338)	(7.140)
cons	275.230**	302.171**	312.175**	276.361**	289.487**	292.770**
	(124.073)	(122.852)	(126.956)	(125.318)	(121.482)	(124.670)
N	264	264	264	265	265	265
R^2	0.056	0.069	0.069	0.057	0.069	0.069
$Prob > F$	0.352	0.187	0.156	0.342	0.167	0.0377

Note. Dependent Variable: *requests* (continuous). Independent Variables: are all refered to monitors' evaluations on subjects' characteristics, *a*: 1 (very thin) to 7 (very obese); *b=a* if $a \geq 5$, 0 otherwise; *dover*: 1 if a>*self-weight*, 0 otherwise; *overmrweight* and *overmrob3* are the interactions between *a, b* and *dover* ; *mr-beauty*:1 (very ugly) to 7 (very beautiful); mr_self-est: 1:(no self-esteem at all) to 7 (high self-esteem); mr_ambition: 1(not ambitious at all) to 7(very ambitious); age, age^2 and $wage$ continuous variables. Standard errors (adjusted for 27 clusters in interviewers) of parameters estimates in parentheses.$^*p < 0.10$, $^{**}p < 0.05$,$^{***}p < 0.01$. Four observations are excluded as outliers (>3*s.d.). (1)-(3): Main regressors mr_weight and *overmrweight*(4)-(6): Main regressors $ob3$ and *overmrob3*

PART 1

In the following questions you are asked to describe your physical characteristics and your personality. Please check the number that describes better the level of the following characteristics:

Regarding your physical characteristics, you consider yourself:

(note that 4 means neither the one nor the other characteristic)

a) ugly :. . . 1. . . 2. . . . . . 3. . . . . . 4. . . . . . 5. . . . . . 6. . . . . . 7. . . handsome/beautiful
(note that 4 means neither ugly nor handsome)

b) thin :. . . 1. . . 2. . . . . . 3. . . . . . 4. . . . . . 5. . . . . . 6. . . . . . 7. . . obese

c) badly dressed :. . . 1. . . 2. . . 3. . . 4 . . . 5. . . 6 . . . 7. . . well-dressed

d) short :. . . 1. . . 2. . . 3. . . 4 . . . 5. . . 6 . . . 7. . . tall

Regarding your personality, you consider yourself:

e) shy :. . . 1. . . 2. . . 3. . . 4 . . . 5. . . 6 . . . 7. . . leader

f) introverted :. . . 1. . . 2. . . 3. . . 4 . . . 5. . . 6 . . . 7. . . very social

g) anodyne :. . . 1. . . 2. . . 3. . . 4 . . . 5. . . 6 . . . 7creative

h) bad person :. . . 1. . . 2. . . 3. . . 4 . . . 5. . . 6 . . . 7. . . nice person

i) no ambitious :. . . 1. . . 2. . . 3. . . 4 . . . 5. . . 6 . . . 7. . . very ambitious

j) no self-esteem :. . . 1. . . 2. . . 3. . . 4 . . . 5. . . 6 . . . 7. . . very self-esteem

PART 3

At this moment, we would like to know the amount of money that you would like to request as a compensation for the effort you made to complete the questionnaire and for the information you provide us. The money disposed for this research project is given by the State. Do not forget that this money does not belong neither to us (neither affect us) nor to the State.

How much money would you like to receive for filling out this questionnaire?

I request the following amount of money: . . . . . . euros

In the attached stick we would like you to fill in your full name and address in order for us to send your money by mail. Obviously, this is optional, but in the case you want to receive your payment it is the only way. Please read the following compromise regarding data protection.

PAPER STICK HERE

Please, provide us with your phone number or e-mail address (or both), in order to contact you in about two weeks time for confirming the reception of the money sent.

Mobile number:

E-mail:

According to the *Law of Data Protection*, the information provided in the previous pages is not going to be corresponded with your personal data. Finally, in

Economics Faculty, there are constantly experiments organized. In these experiments, of various types (on-line, by mail, presence, etc) different types of people participate and of course money are earned depending on participantsperformance on the tasks. If you like it, we can include your personal data in our data base in order to inform you when you can earn some money. In order to be more operative and no annoying you for things that you are not interested in, we ask you to tell us from which amount of money you would be interested in participating.

- Are you interested in participating in one of these? YES. . . . . . NO. . . . . .
- In the case of being interesting, from which amount money would you willing to participate?. . . . . .
- If you had to come to the Faculty of Economics (Faculty's name blinded for peer review) would you do it? YES. . . . . . NO. . . . . .

Thank you very much for your effort and help, authors' names blinded for peer review.

Supplementary Figure 1. Subjects' Questionnare. English translation of Parts 1 and 3.

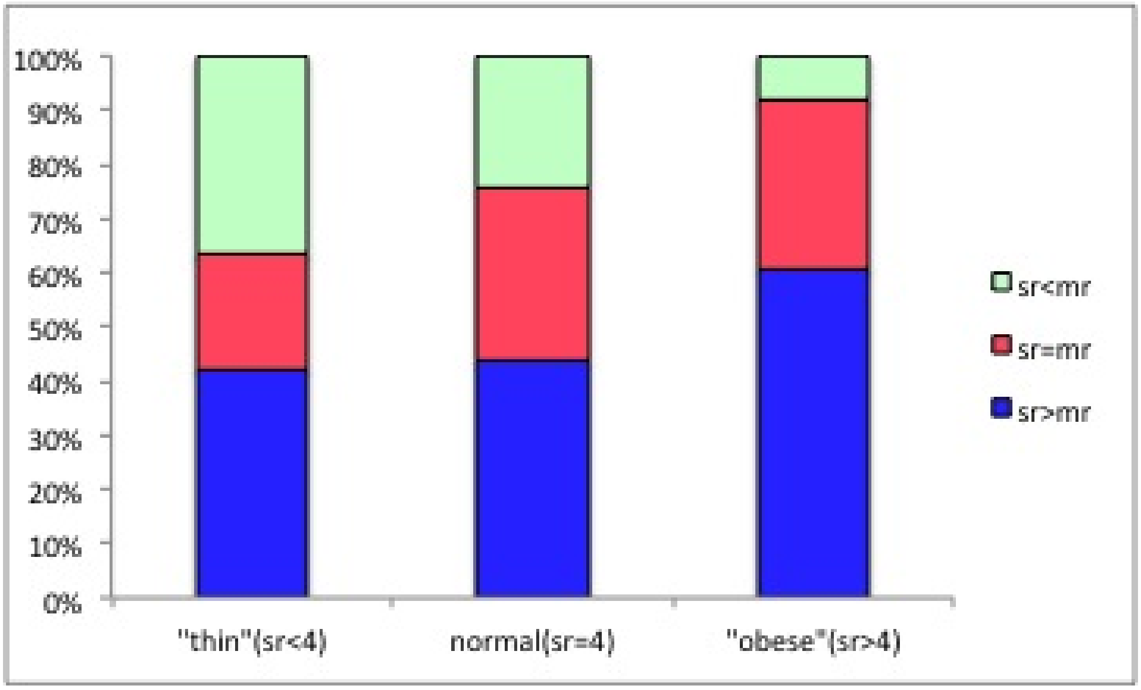

Supplementary Figure 2. Subjects' reports vs. monitors' reports. Percentage of people who understate ($sr < mr$), accurately-state ($sr = mr$) or overstate ($sr > mr$) their self-weight as compared to monitors' evaluations.sr and mr stand for self-reported and monitor-reported weight status.
